# Supplementary material for: Data-Mining-Aided-Material Design of Doped LaMnO3 Perovskites with Higher Curie Temperature
Source: Materials (Basel). 2025 May 23;18(11):2437. doi: 10.3390/ma18112437 (PMC12155942; doi:10.3390/ma18112437)
Supplement: Supplementary file 1 [file materials-18-02437-s001.zip › materials-3609268-supplementary.pdf]

Table S1 The dataset for modeling.

NO.	Formula	Tc	Radius_A	Radius_B	Za	Zb	TF	aO3	rc	A_ionic	B_ionic	R_a/R_b	Mass	A_aff	B_aff	A_Tm	B_Tm	A_Tb	B_Tb	A_Hfus	B_Hfus	A_Density	B_Density
1	La0.7Sr0.3Mn0.5Cr0.5O3	226	107.64	59.75	1.055	1.605	0.877	143.80	61.89	5.612	7.100	1.802	225.0	24.90	32.15	875.70	1576.5	2839.40	2366	56.66	320	5.10	7.23
2	La0.7Sr0.3Mn0.8Cr0.2O3	286	107.64	58.70	1.055	1.572	0.881	141.32	61.39	5.612	7.301	1.834	225.9	24.90	12.86	875.70	1378.2	2839.40	2183	56.66	269	5.10	7.27
3	La0.7Sr0.3Mn0.9Cu0.1O3	350	107.64	57.60	1.055	1.585	0.886	138.73	60.88	5.612	7.463	1.869	227.3	24.90	11.18	875.70	1229.9	2839.40	2111	56.66	232	5.10	7.47
4	La0.75Sr0.25Mn1O3	340	106.90	58.00	1.063	1.550	0.882	139.66	60.83	5.606	7.434	1.843	229.0	28.75	0.00	882.75	1246.0	2943.50	2061	54.65	235	5.27	7.30
5	La0.7Sr0.3Mn0.6Cr0.4O3	242	107.64	59.40	1.055	1.594	0.878	142.97	61.72	5.612	7.167	1.812	225.3	24.90	25.72	875.70	1510.4	2839.40	2305	56.66	303	5.10	7.24
6	La0.7Sr0.25Ag0.05Mn1O3	303	105.49	58.00	1.104	1.550	0.877	139.65	60.39	5.706	7.434	1.819	227.5	32.63	0.00	884.94	1246.0	2878.40	2061	57.65	235	5.49	7.30
7	La0.7Sr0.05Ag0.25Mn1O3	363	96.89	58.00	1.300	1.550	0.846	139.57	57.83	6.083	7.434	1.671	231.5	63.55	0.00	921.90	1246.0	3034.40	2061	61.61	235	7.06	7.30
8	La0.75Ba0.1Ag0.15Mn1O3	315	102.15	58.00	1.204	1.550	0.865	139.62	59.38	5.840	7.434	1.761	237.0	51.94	0.00	905.47	1246.0	3110.20	2061	54.32	235	6.55	7.30
9	La0.7Ca0.3Mn1O3	250	102.24	58.00	1.070	1.550	0.865	139.62	59.40	5.738	7.434	1.763	212.2	24.90	0.00	895.20	1246.0	2870.00	2061	95.15	235	4.77	7.30
10	La0.7Ag0.3Mn1O3	270	94.74	58.00	1.349	1.550	0.838	139.54	57.23	6.177	7.434	1.633	232.5	71.28	0.00	931.13	1246.0	3073.40	2061	62.60	235	7.46	7.30
11	La0.89Sr0.11Mn1O3	195	104.83	58.00	1.084	1.550	0.874	139.64	60.19	5.590	7.434	1.807	236.2	39.53	0.00	902.49	1246.0	3234.98	2061	49.02	235	5.76	7.30
12	La0.88Sr0.12Mn1O3	170	104.98	58.00	1.082	1.550	0.875	139.64	60.24	5.591	7.434	1.810	235.7	38.76	0.00	901.08	1246.0	3214.16	2061	49.42	235	5.73	7.30
13	La0.875Sr0.125Mn1O3	188	105.05	58.00	1.081	1.550	0.875	139.64	60.26	5.592	7.434	1.811	235.4	38.38	0.00	900.38	1246.0	3203.75	2061	49.63	235	5.71	7.30
14	La0.865Sr0.135Mn1O3	214	105.20	58.00	1.080	1.550	0.876	139.65	60.30	5.593	7.434	1.814	234.9	37.61	0.00	898.96	1246.0	3182.93	2061	50.03	235	5.68	7.30
15	La0.855Sr0.145Mn1O3	230.5	105.35	58.00	1.078	1.550	0.876	139.65	60.35	5.594	7.434	1.816	234.4	36.84	0.00	897.56	1246.0	3162.11	2061	50.43	235	5.64	7.30
16	La0.845Sr0.155Mn1O3	242	105.49	58.00	1.077	1.550	0.877	139.65	60.40	5.595	7.434	1.819	233.9	36.07	0.00	896.15	1246.0	3141.29	2061	50.83	235	5.61	7.30
17	La0.835Sr0.165Mn1O3	260.5	105.64	58.00	1.075	1.550	0.877	139.65	60.44	5.596	7.434	1.821	233.4	35.30	0.00	894.74	1246.0	3120.47	2061	51.23	235	5.57	7.30
18	La0.83Sr0.17Mn1O3	265	105.72	58.00	1.075	1.550	0.878	139.65	60.46	5.597	7.434	1.823	233.1	34.91	0.00	894.03	1246.0	3110.06	2061	51.43	235	5.55	7.30
19	La0.825Sr0.175Mn1O3	283	105.79	58.00	1.074	1.550	0.878	139.65	60.49	5.598	7.434	1.824	232.9	34.52	0.00	893.32	1246.0	3099.65	2061	51.64	235	5.54	7.30
20	La0.72Sr0.28Mn1O3	375	107.34	58.00	1.058	1.550	0.883	139.67	60.97	5.610	7.434	1.851	227.5	26.44	0.00	878.52	1246.0	2881.04	2061	55.86	235	5.17	7.30
21	La0.69Sr0.31Mn1O3	380	107.79	58.00	1.054	1.550	0.885	139.67	61.11	5.613	7.434	1.858	225.9	24.13	0.00	874.29	1246.0	2818.58	2061	57.06	235	5.06	7.30
22	La0.64Sr0.36Mn1O3	372	108.53	58.00	1.046	1.550	0.888	139.68	61.35	5.619	7.434	1.871	223.4	20.28	0.00	867.24	1246.0	2714.48	2061	59.07	235	4.89	7.30
23	La0.52Sr0.48Mn1O3	330	110.30	58.00	1.028	1.550	0.894	139.69	61.91	5.634	7.434	1.902	217.2	11.04	0.00	850.32	1246.0	2464.64	2061	63.90	235	4.47	7.30
24	La0.50Sr0.50Mn1O3	310	110.60	58.00	1.025	1.550	0.895	139.70	62.01	5.636	7.434	1.907	216.2	9.50	0.00	847.50	1246.0	2423.00	2061	64.70	235	4.40	7.30

25	La0.48Sr0.52Mn1O3	290	110.90	58.00	1.022	1.550	0.896	139.70	62.11	5.638	7.434	1.912	215.2	7.96	0.00	844.68	1246.0	2381.36	2061	65.50	235	4.32	7.30
26	La0.45Sr0.55Mn1O3	260	111.34	58.00	1.018	1.550	0.898	139.70	62.25	5.642	7.434	1.920	213.6	5.65	0.00	840.45	1246.0	2318.90	2061	66.71	235	4.22	7.30
27	La0.4Sm0.3Sr0.3Mn1O3	256	105.42	58.00	1.076	1.550	0.876	139.65	60.37	5.632	7.434	1.818	229.9	24.60	0.00	922.50	1246.0	2338.40	2061	60.47	235	5.51	7.30
28	La0.7Sr0.3Mn0.93Fe0.07O3	296	107.64	57.79	1.055	1.570	0.885	139.17	60.97	5.612	7.467	1.863	226.5	24.90	1.10	875.70	1266.4	2839.40	2117	56.66	236	5.10	7.34
29	La0.7Sr0.3Mn0.9Al0.1O3	310	107.64	57.53	1.055	1.556	0.886	138.56	60.85	5.612	7.289	1.871	223.7	24.90	4.26	875.70	1187.4	2839.40	2107	56.66	251	5.10	6.84
30	La0.67Ca0.33Mn0.85V0.15O3	287.2	102.14	58.90	1.067	1.562	0.861	141.74	59.82	5.754	7.331	1.734	208.6	22.59	7.61	892.92	1345.6	2810.60	2263	100.21	263	4.63	7.11
31	La0.6Nd0.1Ca0.15Sr0.15Mn0.9Fe0.1O3	298	104.45	57.70	1.067	1.578	0.874	138.93	59.93	5.670	7.481	1.810	219.9	24.80	1.57	895.75	1275.2	2815.70	2141	76.40	236	5.02	7.36
32	La0.6Nd0.1Ca0.15Sr0.15Mn1O3	326	104.45	58.00	1.067	1.550	0.873	139.64	60.07	5.670	7.434	1.801	219.9	24.80	0.00	895.75	1246.0	2815.70	2061	76.40	235	5.02	7.30
33	La0.6Nd0.1Ca0.15Sr0.15Mn0.95Fe0.05O3	306	104.45	57.85	1.067	1.564	0.874	139.29	60.00	5.670	7.457	1.806	219.9	24.80	0.79	895.75	1260.6	2815.70	2101	76.40	236	5.02	7.33
34	La0.9Pb0.1Mn1O3	235	104.78	58.00	1.177	1.550	0.874	139.64	60.18	5.761	7.434	1.807	248.7	46.71	0.00	858.95	1246.0	3292.50	2061	42.45	235	6.67	7.30
35	La0.8Pb0.2Mn1O3	310	106.36	58.00	1.254	1.550	0.880	139.66	60.67	5.945	7.434	1.834	255.5	45.42	0.00	799.89	1246.0	3121.00	2061	40.30	235	7.18	7.30
36	La0.7Pb0.3Mn1O3	358	107.94	58.00	1.331	1.550	0.885	139.67	61.16	6.129	7.434	1.861	262.3	44.13	0.00	740.84	1246.0	2949.50	2061	38.15	235	7.70	7.30
37	La0.6Pb0.4Mn1O3	360	109.52	58.00	1.408	1.550	0.891	139.69	61.66	6.313	7.434	1.888	269.2	42.84	0.00	681.78	1246.0	2778.00	2061	36.00	235	8.21	7.30
38	La0.5Pb0.5Mn1O3	355	111.10	58.00	1.485	1.550	0.897	139.70	62.17	6.497	7.434	1.916	276.0	41.55	0.00	622.73	1246.0	2606.50	2061	33.85	235	8.73	7.30
39	La0.65Sr0.35Mn1O3	377	108.38	58.00	1.048	1.550	0.887	139.68	61.30	5.618	7.434	1.869	223.9	21.05	0.00	868.65	1246.0	2735.30	2061	58.67	235	4.92	7.30
40	La0.55Pr0.1Sr0.35Mn1O3	353	107.96	58.00	1.051	1.550	0.886	139.67	61.17	5.608	7.434	1.861	224.1	20.95	0.00	869.95	1246.0	2740.90	2061	59.10	235	4.98	7.30
41	La0.45Pr0.2Sr0.35Mn1O3	344	107.54	58.00	1.054	1.550	0.884	139.67	61.04	5.597	7.434	1.854	224.3	20.85	0.00	871.25	1246.0	2746.50	2061	59.53	235	5.05	7.30
42	La0.35Pr0.3Sr0.35Mn1O3	334	107.12	58.00	1.057	1.550	0.883	139.66	60.90	5.587	7.434	1.847	224.5	20.75	0.00	872.55	1246.0	2752.10	2061	59.96	235	5.11	7.30
43	La0.7Sr0.1Ag0.2Mn1O3	286.5	99.04	58.00	1.251	1.550	0.854	139.59	58.45	5.989	7.434	1.708	230.5	55.82	0.00	912.66	1246.0	2995.40	2061	60.62	235	6.67	7.30
44	La0.67Sr0.33Mn1O3	372.5	108.08	58.00	1.051	1.550	0.886	139.67	61.21	5.616	7.434	1.864	224.9	22.59	0.00	871.47	1246.0	2776.94	2061	57.87	235	4.99	7.30
45	La0.7Sr0.3Mn1O3	370	107.64	58.00	1.055	1.550	0.884	139.67	61.07	5.612	7.434	1.856	226.5	24.90	0.00	875.70	1246.0	2839.40	2061	56.66	235	5.10	7.30
46	La0.7Sr0.3Mn0.95Fe0.05O3	330	107.64	57.85	1.055	1.564	0.885	139.31	61.00	5.612	7.457	1.861	226.5	24.90	0.79	875.70	1260.6	2839.40	2101	56.66	236	5.10	7.33
47	La0.7Sr0.3Mn0.9Cr0.1O3	326	107.64	58.35	1.055	1.561	0.883	140.49	61.23	5.612	7.367	1.845	226.2	24.90	6.43	875.70	1312.1	2839.40	2122	56.66	252	5.10	7.29
48	La0.7Sr0.3Mn0.85Cr0.15O3	304	107.64	58.53	1.055	1.566	0.882	140.91	61.31	5.612	7.334	1.839	226.0	24.90	9.65	875.70	1345.1	2839.40	2153	56.66	260	5.10	7.28
49	La0.7Sr0.3Mn0.85Fe0.15O3	175	107.64	57.55	1.055	1.592	0.886	138.61	60.86	5.612	7.504	1.870	226.6	24.90	2.36	875.70	1289.8	2839.40	2181	56.66	237	5.10	7.39
50	La0.68Nd0.02Ba0.3Mn0.9Cr0.1O3	300	112.64	58.35	1.038	1.561	0.901	140.54	62.83	5.466	7.367	1.930	241.2	24.88	6.43	862.76	1312.1	2980.70	2122	46.86	252	5.41	7.29

51	La0.7Ba0.3Mn0.9Cr0.1O3	298	112.74	58.35	1.037	1.561	0.901	140.54	62.86	5.467	7.367	1.932	241.1	24.90	6.43	860.70	1312.1	2988.50	2122	46.76	252	5.39	7.29
52	La0.7Sr0.3Mn0.9Fe0.1O3	261	107.64	57.70	1.055	1.578	0.886	138.96	60.93	5.612	7.481	1.866	226.5	24.90	1.57	875.70	1275.2	2839.40	2141	56.66	236	5.10	7.36
53	La0.7Ba0.3Mn0.9Fe0.1O3	215	112.74	57.70	1.037	1.578	0.904	139.01	62.57	5.467	7.481	1.954	241.5	24.90	1.57	860.70	1275.2	2988.50	2141	46.76	236	5.39	7.36
54	La0.67Ca0.33Mn1O3	275	102.14	58.00	1.067	1.550	0.865	139.62	59.37	5.754	7.434	1.761	209.2	22.59	0.00	892.92	1246.0	2810.60	2061	100.21	235	4.63	7.30
55	La0.6Sr0.1Cu0.3Mn1O3	232	89.92	58.00	1.325	1.550	0.821	139.49	55.92	6.234	7.434	1.550	214.1	59.44	0.00	953.89	1246.0	2985.20	2061	96.29	235	6.64	7.30
56	La0.65Ca0.18Sr0.17Mn1O3	323	105.14	58.00	1.057	1.550	0.875	139.65	60.29	5.693	7.434	1.813	215.3	21.05	0.00	880.35	1246.0	2753.66	2061	81.76	235	4.72	7.30
57	La0.67Ba0.33Mn0.98Ti0.02O3	314	113.69	58.18	1.031	1.550	0.905	140.15	63.10	5.456	7.422	1.954	241.2	22.59	0.15	854.97	1254.5	2940.95	2086	46.98	236	5.32	7.24
58	La0.7Ca0.2Sr0.1Mn1O3	315	104.04	58.00	1.065	1.550	0.872	139.64	59.95	5.696	7.434	1.794	216.9	24.90	0.00	888.70	1246.0	2859.80	2061	82.32	235	4.88	7.30
59	La0.65Nd0.05Ca0.3Mn1O3	250	102.00	58.00	1.072	1.550	0.864	139.62	59.33	5.735	7.434	1.759	212.5	24.85	0.00	900.35	1246.0	2850.50	2061	95.40	235	4.81	7.30
60	La0.6Sr0.2Ba0.2Mn1O3	354	112.52	58.00	1.028	1.550	0.902	139.71	62.63	5.527	7.434	1.940	231.3	17.20	0.00	851.60	1246.0	2730.60	2061	54.08	235	4.94	7.30
61	La0.8Ba0.1Ca0.1Mn0.97Fe0.03O3	281	106.06	57.91	1.069	1.558	0.879	139.44	60.53	5.594	7.448	1.831	231.8	32.60	0.47	891.30	1254.8	3107.50	2085	62.17	235	5.44	7.32
62	La0.9Mg0.1Mn1O3	160	100.08	58.00	1.121	1.550	0.857	139.60	58.76	5.784	7.434	1.726	230.4	39.30	0.00	891.20	1246.0	3226.60	2061	75.03	235	5.71	7.30
63	La0.8Ba0.2Mn1O3	295	109.56	58.00	1.058	1.550	0.891	139.69	61.68	5.504	7.434	1.889	241.5	32.60	0.00	879.80	1246.0	3147.00	2061	46.04	235	5.64	7.30
64	La0.67Ba0.23Ca0.1Mn1O3	350	110.19	58.00	1.042	1.550	0.894	139.69	61.88	5.547	7.434	1.900	231.6	22.59	0.00	866.47	1246.0	2901.45	2061	63.11	235	5.11	7.30
65	La0.7Ba0.3Mn1O3	328	112.74	58.00	1.037	1.550	0.903	139.71	62.70	5.467	7.434	1.944	241.4	24.90	0.00	860.70	1246.0	2988.50	2061	46.76	235	5.39	7.30
66	La0.57Dy0.1Sr0.33Mn1O3	358	106.88	58.00	1.063	1.550	0.882	139.66	60.83	5.652	7.434	1.843	227.3	22.49	0.00	920.87	1246.0	2687.24	2061	60.22	235	5.23	7.30
